# Construction of the genetic switches in response to mannitol based on artificial MtlR box

**DOI:** 10.1186/s40643-023-00634-7

**Published:** 2023-01-30

**Authors:** Fengxu Xiao, Yupeng Zhang, Liang Zhang, Zhongyang Ding, Guiyang Shi, Youran Li

**Affiliations:** 1 Key Laboratory of Industrial Biotechnology, Ministry of Education, School of Biotechnology, Jiangnan University, Wuxi, 214122 People’s Republic of China; 2 National Engineering Research Center for Cereal Fermentation and Food Biomanufacturing, Jiangnan University, 1800 Lihu Avenue, Wuxi, 214122 Jiangsu People’s Republic of China; 3 Jiangsu Provincial Engineering Research Center for Bioactive Product Processing, Jiangnan University, Wuxi, 214122 Jiangsu People’s Republic of China

**Keywords:** Genetic switch, MtlR box, Cre site, *Bacillus licheniformis*

## Abstract

Synthetic biology has rapidly advanced from the setup of native genetic devices to the design of artificial elements able to provide organisms with highly controllable functions. In particular, genetic switches are crucial for deploying new layers of regulation into the engineered organisms. While the assembly and mutagenesis of native elements have been extensively studied, limited progress has been made in rational design of genetic switches due to a lack of understanding of the molecular mechanism by which a specific transcription factor interacts with its target gene. Here, a reliable workflow is presented for designing two categories of genetic elements, one is the switch element-MtlR box and the other is the transcriptional regulatory element- catabolite control protein A (CcpA) box. The MtlR box was designed for ON/OFF-state selection and is controlled by mannitol. The rational design of MtlR box-based molecular structures can flexibly tuned the selection of both ON and OFF states with different output switchability in response to varied kind effectors. Different types of CcpA boxes made the switches with more markedly inducer sensitivities. Ultimately, the OFF-state value was reduced by 90.69%, and the maximum change range in the presence of two boxes was 15.31-fold. This study presents a specific design of the switch, in a plug-and-play manner, which has great potential for controlling the flow of the metabolic pathway in synthetic biology.

**Graphical Abstract:**

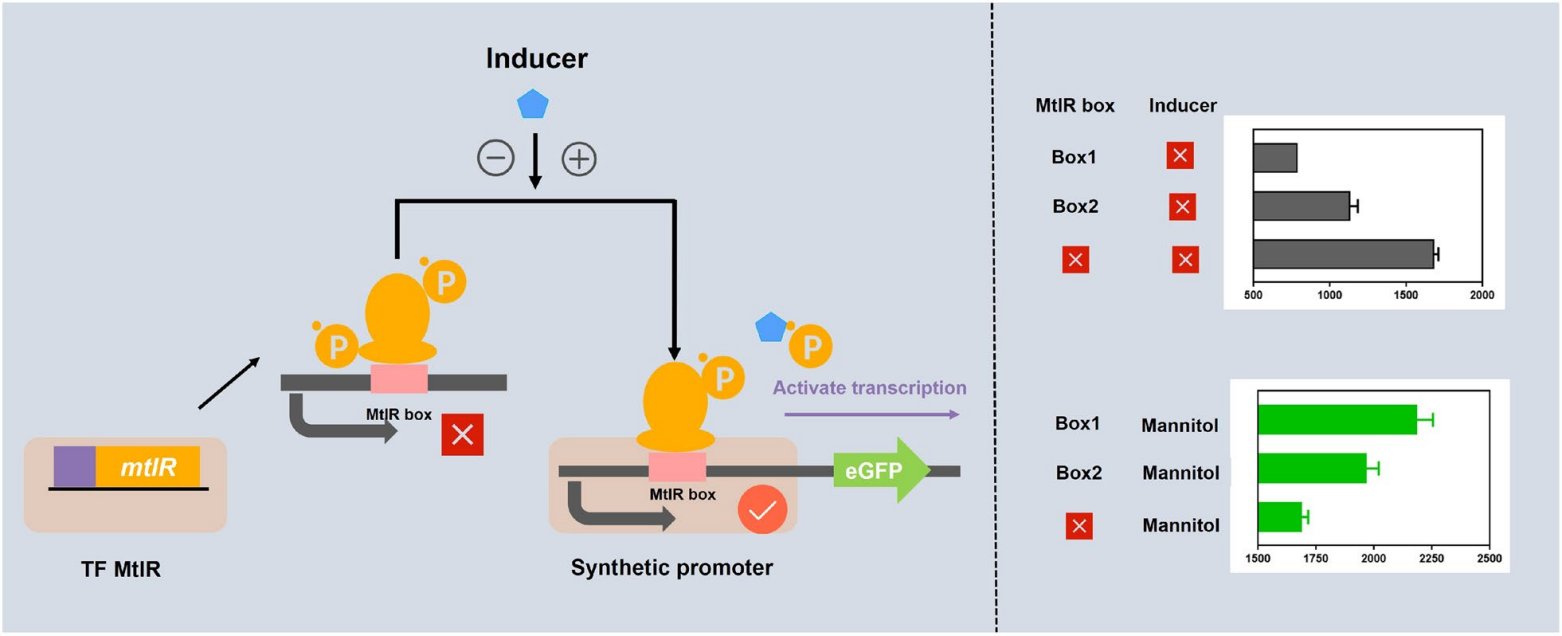

**Supplementary Information:**

The online version contains supplementary material available at 10.1186/s40643-023-00634-7.

## Introduction

The switch is a fundamental component that has been widely applied into complex systems in fields which ranging from electronic engineering to engineering biology. The modular and orthogonal genetic switches are critical for constructing a gene circuit that robustly controls the gene expression in synthetic biology (Wang et al. 2011). Commonly, a genetic switch was composed of a repressor or activator, a targeting DNA sequence and an effector. These pieces worked together to achieve the switching of the ON-state and OFF-state. The switching of the state was guided by diffusible signaling molecules such as light (Kong et al.^2014^), compound (Seo et al. 2019) and quorum sensing (Rajput et al. 2016). Engineered genetic circuits that carry switches have been used to design biosensors (Zhang et al. 2021), screen for drugs (Xie et al. 2020), product high-value chemicals and biocomputing (Inniss and Silver 2013; Wu et al. 2020). The topology of the genetic circuits that carry switches are usually composed of two cross-repressing nodes, resulting in a node expression under the binary mutually exclusive model (Perez-Carrasco et al. 2016). If the switch was affected by ‘outside-in’ signaling, the TFs would affect responsive gradient to output of this signal through fluctuation of the gene expression. Living organisms have multiple types of switches that control their behaviors including feed-forward loops (Schaerli et al. 2014), inducible promoters (Kluge et al. 2018) and AND gates (Boehm et al. 2018). Since the native switches have a limited dynamic ranges and create output noise, the switch selection involves a transition from native genetic devices to programmed designs (Du et al. 2021). To date, changing the preference of TFs for a DNA sequence by remodeling TF DNA binding domains has been demonstrated to be an effective method of amending the parameters of the switch (Gaber et al. 2014). One example is the TALE protein, which was initially found in phytopathogenic bacteria. This protein has been favored by researchers because of its programmable DNA binding domains (Garg et al. 2012). The TALE protein DNA binding domain contains different numbers of amino acid repetitions, which can bind to any specific DNA sequence through DNA binding recombination (Mak et al. 2012). Since TFs recognition sites exist in the endogenous genes by means of a collection of a set of similar DNA sequences, the system may be subjected to extra interference caused by changing of TFs DNA binding domains, which introduces a new variable of the modular processes. Notably, the design of the switch should follow the principle with minimal negative impact on the host cell. Usually, it requires a group of specially programmed DNA sequences to assemble a genetic circuit. With this programmed DNA sequence, cellular behavior is not only controlled by the switch, but also has a reduced effect on the cells. The research of the switch was delayed, as it was limited by the number of the programmed DNA sequences. Some TFs could be applied to the switch, whereas lacked programmed DNA sequence (Eggeling 2018). In addition, another obstacle in designing the programmed DNA sequence is a blurry molecular mechanism for a specific transcription factor. Hence, the key of constructing genetic switches is a set of reliable programmed DNA sequences.

A vital step of switch design is TFs selection. The current switch state is related to the input signal. If the switch is in the OFF-state with the input signal, it switches to the ON-state and vice versa (Barbier et al. 2020). MtlR protein was expected to be one of candidates for TFs in genetic switches. MtlR protein has been confirmed as a transcription factor for regulating the expression of structural genes (*mtlA*, *mtlD*, *mtlF*) in *mtl* operon (Xiao et al. 2020). MtlR protein was made up of three different regions (1) an N-terminal DNA binding region (2) a central domain consisting of two PTS regulation domains (PRDs) (3) a C-terminal domain containing an EIIB(Gat) and EIIA (Mtl)-like domain (Joyet et al. 2010). Phosphorylation/dephosphorylation of the MtlR protein’s two PRDs are the key steps of switching the inducer signal (Bouraoui et al. 2013). In the absence of mannitol or other substrate, the MtlR protein’s two PRDs were phosphorylated by Hpr and IIBmtl, respectively. These processes made a low affinity of MtlR for the MtlR box and blocked mannitol operon structural gene expression. In the presence of mannitol, the phosphoryl group from the IICB is transferred to mannitol. In this way, PRD-II dephosphorylation leads to a high affinity to MtlR for the MtlR box and activates the mannitol operon structural gene expression (Henstra et al. 1999). In the presence of the input signal, the state of the MtlR protein was adjusted accordingly and the output value was also changed. Therefore, a switch was constructed by mannitol, MtlR protein, and the MtlR box. Regrettably, lacking the study of MtlR box restricts applications in genetic switch. Genetic switch elements like the PdhR box can assemble to different synthetic circuits according to such a ‘plug-and-play’ flexible method (Xu et al. 2020). With the development of synthetic biology, higher requirements have been put forward for genetic switch such as lower basal expression or higher dynamic range. The lower basal expression is beneficial to distinguish the output signal from background interference and the high dynamic range is desirable in gene circuits for dynamic pathway regulation. Combinations of multigroup elements also may be an effective way to enhance the switch performance.

To solve the problems, we attempted to add extra molecular elements to a genetic switch. Since the catabolism and anabolism of carbohydrate was regulated by CcpA, the CcpA box could be brought into the genetic switch. CcpA, a protein of the LacI-GalR family, has been confirmed as a transcription factor for the carbon catabolite activation (CCA) and carbon catabolite repression (CCR) in *Bacillus* (Kraus et al. 2010). Usually, CcpA executes its regulation through binding to the catabolite responsive element (cre) site (Lorca et al. 2005). The sequence of cre was identified as ‘TGWNANCGNTNWCA’ in *Bacillus*, in which N represents any base and W represents A or T (Weickert et al.^1990^). The sequence of two other cre sites were identified as ‘AGCTTT-N-AAAGCT’ and ‘AAAGCT-N-AGCTTT’ in *Bacillus licheniformis*, in which N stands for any bases with different number (Xiao et al. 2021). Such regulatory effects ensure the preferential utilization of glucose in mixed carbon sources. Frequently, the genes that are involved in the transport and catabolism of other carbohydrates would be repressed when glucose is present (Langa et al. 2021). This process was achieved by CcpA-cre. Hence, the cre site was expected to a tool in synthetic biology for expanding circuit dynamic range. In this study, genetic switch was built and tested in *B.licheniformis*. *B.licheniformis* has been regarded as a type of ‘generally recognized as safe’ (GRAS) microorganism and used as a production platform for some high-value enzymes and high-value compounds such as: amylase (Li et al. 2018), peptidases (Wu et al. 2022), poly-γ-glutamic acid (Yu et al. 2017) and 2,3-butanediol (Huo et al. 2018). In this study, we selected MtlR box as a switch element and cre site as the transcription regulatory element. This study demonstrates design and application of two sets of elements that do not be interfered by each other. The logic of design also applies to other carbon responsive boxes.

In this study, a constitutive promoter Pshutle09 was selected as the vehicle to carry these two elements. Two products (eGFP and maltotriose amylase) were used to test the effect of the two elements. The MtlR box was regulated by the repressor protein MtlR and the cre was regulated by the global regulatory factor CcpA. With the combination of the two elements, a genetic circuit with low basial expression and broad dynamic ranges was obtained. This study aimed at designing a mannitol genetic switch for adapting multiple synthetic biology scenes such as substrate-dependent genetic circuit, biosensor, etc. This idea will provide useful information for the gene circuits under complex environments.

## Materials and methods

### Bacterial strains and reagents

Additional file 1: Table S1 lists the strains and plasmids used and constructed in this study. *Escherichia coli* JM109 was used for plasmids construction, and *B.licheniformis* B1391 was used for functional characterization. The positive transformers of *Escherichia coli* harboring recombinant plasmids based on pHY-PLK300 were grown on solid Luria–Bertani (LB) agar broth with 1.5% concentration and selected using 100 µg/mL ampicillin resistance screening. The *B.licheniformis* B1391 transformants were grown on solid Luria–Bertani (LB) agar broth with 1.5% concentration, and selected using 20 µg/mL tetracycline resistance screening. The LB medium (10 g/L tryptone, 5 g/L yeast extract, 10 g/L NaCl) was used for the seed culture of *E.coli* and *B.licheniformis*. The former was cultured at 37 °C and 200 rpm and the latter was cultured at 37 °C and 250 rpm. The effect evaluation of genetic circuit was evaluated using a medium (12 g/L tryptone, 24 g/L yeast extract, 16.427 g/L K_2_HPO_4_.3H_2_O, 2.31 g/L KH_2_PO_4_). The seed liquid was added to the medium with a 3% inoculation.

### Plasmid construction

In this study, high-fidelity polymerase (Vazyme Biotech Co., Ltd. 2 × Phanta Master Mix, China) was used for a polymerase chain reaction (PCR) experiment. The DNA fragments were purified using DNA purification kits (Vazyme Biotech Co., FastPure^®^ Gel DNA Extraction Mini Kit, China). The plasmids were extracted with a plasmid extraction kit (Vazyme Biotech Co., FastPure^®^ Plasmid Mini Kit, China). The restriction enzymes and T4 DNA ligase were purchased from Takara (Takara Bio, Dalian, China). The homologous recombination kit was purchased form Vazyme, and used for plasmid construction. Additional file 1: Table S2 lists the primers in this study, and Additional file 1: Table S3 lists the sequences of artificial promoters.

The plasmid pE that carried eGFP was constructed in a previous study. The plasmid pE was linearized by the restriction enzymes *Hin*dIII and *Xho*I. Taking the construction of the plasmid pPSA4E (MtlR box 4) as an example, the method for inserting MtlR box was the following. The fragments SA4-1 and SA4-2 were cloned using the fragment Pshutle09 as template and the primer pairs Pshutle09-F/SA4-R and SA4-F/Pshutle09-R, respectively. Then, the fragment A-4 was amplified using SA4-1 and SA4-2 as template and primer pair Pshutle09-F/Pshutle09-R. Finally, the fragment A-4 ligated with the linearized plasmid pE through homologous recombination, generating the pPSA4E plasmid. Using the same method as pPSA4E plasmid, the plasmids pPSAE, pPSBE, pPSA1E, pPSA2E, pPSA3E, pPSA5E, and pPSA6E were constructed.

Taking the construction of the plasmid pPSA4C5E (MtlR box 4, cre3) as an example, the method for adding cre site was the following. The fragments SA4C5-1 and SA4C5-2 were cloned using the fragment A-4 as template and the primer pairs Pshutle09-F/SA4C5-R and SA4C5-F/Pshutle09-R, respectively. Then, the fragment A-4e was amplified using SA4C5-1 and SA4C5-2 as template and primer pair Pshutle09-F/Pshutle09-R. Finally, the fragment A-4e ligated with the linearized plasmid pE through homologous recombination, generating the pPSA4C5E plasmid. Using the same method as pPSA4C5E plasmid, the plasmids pPSA4C1E, pPSA4C2E, pPSA4C3E, pPSA4C4E, pPSA4C6E were constructed.

Taking the construction of the plasmid pSASMAT as an example, the method for carrying MA was the following. Gene synthesis and codon optimization was performed for MA (Additional file 1: Table S3). The maltotriose amylase gene (MA) was cloned using MA as template and primer pair MA-F/MA-R. Then, the fragment was purified and digested with *Kpn*I and *Sal*I, and ligated with the linearized plasmid pHY-PLK300, generating the plasmid pMA. The terminator was cloned by T-F/T-R, and digested with *Sal*I and *Sma*I, then ligated with the linearized plasmid pMA, generating the plasmid pMAT. The signal peptide was cloned by SP-F/SP-R, and digested with *Bam*HI and *Kpn*I, and ligated with linearized plasmid pMAT, generating plasmid pSMAT. The promoter A-4c was cloned using the fragment A-4c as template and the primer pair Pshutle09-F2/Pshutle09-R2. Then, A-4c was digested with *Hin*dIII and *Bam*HI. Ultimately, the plasmid pSASMAT was generated using A-4c ligated with the linearized plasmid pSMAT. According to the same methods, the Pshutle09 was ligated with the linearized plasmid pSMAT, generating plasmid pSSMAT.

### Culture conditions

To test the effect of the MtlR box, the strains that carried different MtlR boxes were activated in an LB agar broth, and a single colony on the LB medium was selected to be activated overnight. The seed liquid was inoculated into the fermentation medium (12 g/L tryptone, 24 g/L yeast extract, 16.427 g/L K_2_HPO_4_.3H_2_O, 2.31 g/L KH_2_PO_4_) with a 3% inoculum, and the 1.5% mannitol or sorbitol was added at 6 h. The sample underwent tests of biomass and fluorescence intensity at 18 h. To test the effect of the cre box, two carbons (1.5% mannitol and 1.5% glucose) were added at 6 h. The sample was observed for tests of biomass and fluorescence intensity at 18 h. To test the application of the MtlR box and cre in enzyme production, the strains that carried pSMAT or pSSMAT were activated in LB agar broth, and a single colony of LB medium was selected to be activated overnight. The seed liquid was inoculated into the fermentation medium (30 g/L glucose, 20 g/L tryptone, 10 g/L yeast extract, 10 g/L (NH_4_)_2_HPO_4_, 9.12 g/L K_2_HPO_4_.3H_2_O, 1.36 g/L KH_2_PO_4_, 0.5 g/L CaCl_2_, and 0.5 g/L MgSO_4_.7H_2_O) with a 3% inoculum, and the 1.5% mannitol was added at 6 h. The sample was observed for tests of biomass and enzyme activity after added inducer 12 h.

### Fluorescence intensity determination

The samples were detached and centrifuged to obtain cell precipitate. Then, the cells were washed with pH 7.4 phosphate buffered saline (PBS) solution twice. Finally, the concentration of cells were diluted to a concentration of 0.5 at OD600. 200 µL of a post-treatment sample was added to the 96-well plate. A TECAN-SparK plate reader (Tecan, Männedorf, Switzerland) was used to conduct fluorescence tests. The program was set to an absorption wavelength of 485 nm, an excitation wavelength of 535 nm, and a gain value of 100.

### Determination of maltotriose amylase activity

The maltotriose amylase activity assay method was based on those of Kim (Kim et al. 1992). One unit of maltotriose amylase was defined as the amount of enzyme required to generate 1 μM of reducing sugar per hour.

### Electrophoretic mobility shift assays

In this study, the MtlR protein and CcpA protein were purified and freeze dried. The DNA probes were amplified using biotin-labeled primer. The DNA probes were purified through gel-cut recovery. The reaction systems involved 10 nM of biotin-labeled DNA probes incubated with different concentrations of MtlR or CcpA in a binding buffer (10 mM Tris–HCl (pH 7.4), 1 mM DTT, 1 mM EDTA, 50 mM KCl, 0.05 µg/µL poly (dI-dC), 1 mM MgCl_2_) under a constant temperature (25 ℃) for 20 min. The probes were separated in 4% acrylamide gels in 0.5 × Tris–borate EDTA (TBE) buffer. Then, the probes were shifted into a nylon membrane (Beyotime, FFN15) and fixed using UV cross-linking. Next, subsequent processing of the nylon membrane was constructed according to the manufacturing protocol with a Chemiluminescent EMSA Kit (Beyotime, GS009).

### Statistical analyses

The results were present as means ± standard deviations (SDs). A Student’s t test was performed for statistical analyses.

## Results and discussion

### Development of mannitol-responsive genetic circuit

**Fig. 1:**
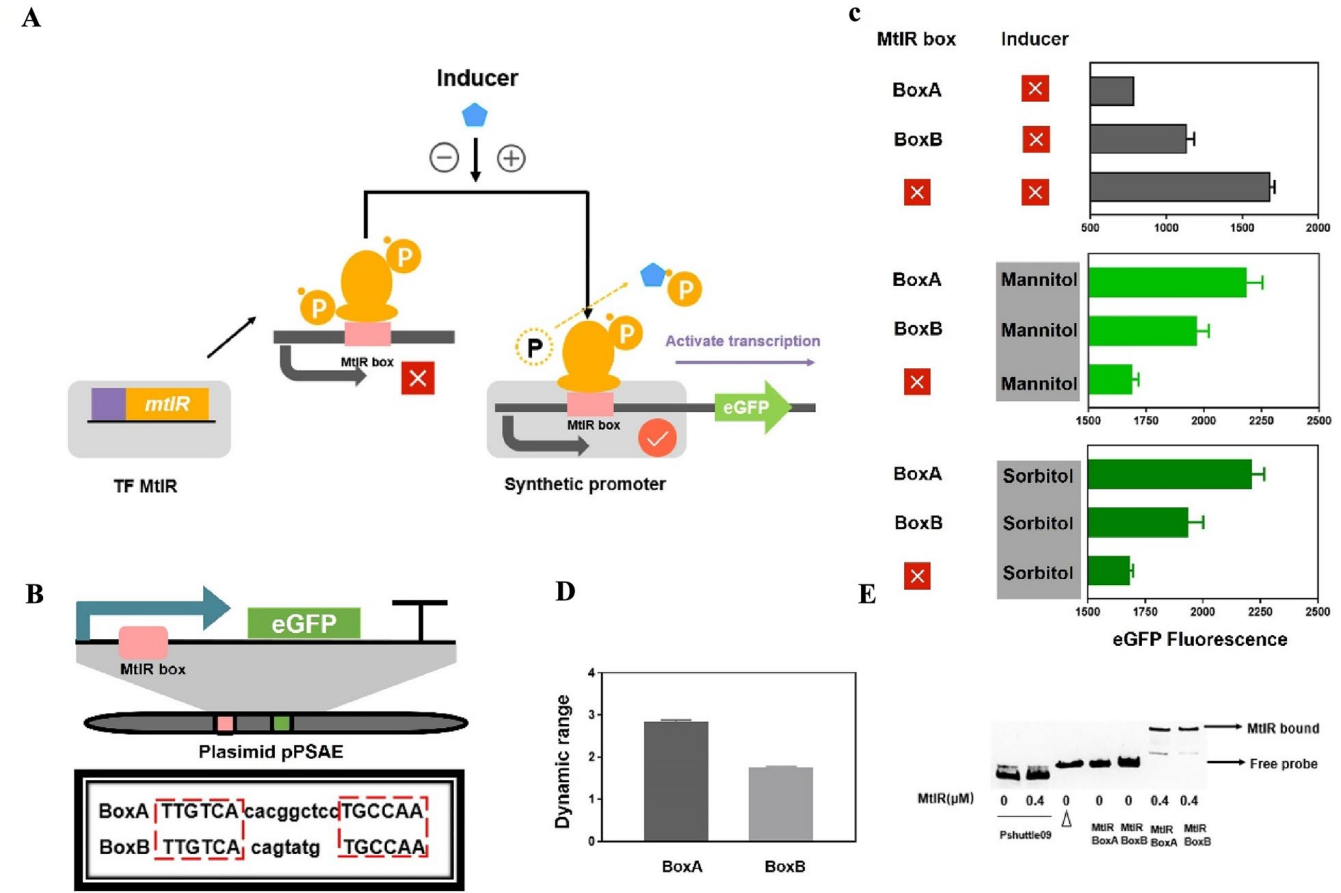
The workflow for designing *B.licheniformis* genetic switches. **A** The design flow of genetic circuit was shown in figure, the inducer controls the indicated OFF/ON selections. OFF selection: in the absence of inducer, MtlR protein’s two PRDs were phosphorylation, made a low affinity for MtlR box, and blocked the expression of target gene. ON selection: in the presence of inducer, the PRD-II region that involved in the negative regulation is dephosphorylated, made a high affinity for MtlR box, then active the expression of target gene. **B** The sequences of MtlR box A and MtlR box B. MtlR box A, originated from *B.licheniformis*, sequence is ‘TTGTCAcacggctccTGCCAA’; MtlR box B, originated from *B.subtilis*, sequence is ‘TTGTCAcagtatgTGCCAA’. And the elements of eGFP expression cassette was shown. **C** The test of genetic switch- MtlR box in vivo. Three strains, BlpPSE, BlpPSAE (contain MtlR Box A), BlpPSBE (contain MtlR Box B) were cultured under three conditions (1. No inducer 2. + 1.5% mannitol 3. + 1.5% sorbitol). The eGFP fluorescence was test after added inducer 12 h. **D** The dynamic range of MtlR box A and MtlR box B. The dynamic ranges of BlpPSAE (contain MtlR Box A), BlpPSBE (contain MtlR Box B) were calculated. The dynamic range was defined as “ON value/ OFF value’. **E** Three fragments (Pshutle09, Pshutle09A, Pshutle09B) were selected as probes, EMSA was performed by MtlR protein in different concentration (0, 0.4 µM) for three fragments

Designable modular TF boxes provide a powerful enrichment toolbox for refining regulation of the biological process (Eggeling 2018; Huang et al. 2019). The majority of TF recognition sites are not a set of the fixation sequence but rather a collection of a set of similar DNA sequences, which have great potential to make genetic circuits for increasing complexity and orthogonality. To develop the mannitol-responsive genetic circuit, the mannitol repressor protein MtlR from *B.licheniformis* was expressed in a heterologous manner in *E.coli*. Using the His-tagged protein kit, the MtlR protein was purified (Additional file 1: Fig. S1). The predicted isoelectric point of MtlR was 6.25, and the predicted molecular mass was 78.14 kDa according to the website (http://web.expasy.org/computer_pi). As shown in Fig. 1A, the ON/OFF-state of genetic circuit was induced by mannitol.

To explore programmable MtlR box in mannitol switch, two boxes (box A, box B) originated from different *Bacillus* were selected as the initial study subjects (Fig. 1B).

Two boxes were inserted into the − 35 region’s upstream of the promoter Pshutle09 separately, thereby generating two artificial promoters named Pshutle09A and Pshutle09B (Additional file 1: Table S4). First, we tested two boxes in vivo from three aspects: (1) the initial value (OFF value) (2) the response value (ON value) (3) the dynamic range. Under the OFF-state, the values of the two artificial genetic circuits (contains box A or box B) were, respectively, decreased 53.28% and 32.85% in base-line, indicating MtlR box blocked the genetic circuit. When mannitol or sorbitol was added, the state of genetic circuit transformed to ON. Under the ON-state (with mannitol), the maximum signal output of two artificial genetic circuits was improved 29.57% and 16.74% comparing to native genetic circuit, respectively (Fig. 1C). When sorbitol adding, the maximum signal output of two artificial genetic circuits was improved 31.56% and 15.02%, respectively (Fig. 1C). Since box A has lower OFF-state value and higher ON-state values, it has a high dynamic range (2.82 ×) (Fig. 1D). Next, we tested two boxes in vitro. To explore the affinity of MtlR for MtlR box, the electrophoretic mobility shift assay (EMSA) experiments was performed. As shown in Fig. 1E, no shifted band was observed when Pshutle09 was used as the probe. This suggested that MtlR had no affinity to Pshutle09. In contrast, when used Pshutle09A or Pshutle09B as probe, the shifted band can be observed. This indicated that MtlR can bind to Pshutle09A or Pshutle09B. Both box A and box B can switch the OFF-state and ON-state for genetic circuits. Then, the molecular structure of MtlR box was investigated to design an ideal genetic switch.

### Rational design of genetic circuits based on the MtlR box

**Fig. 2:**
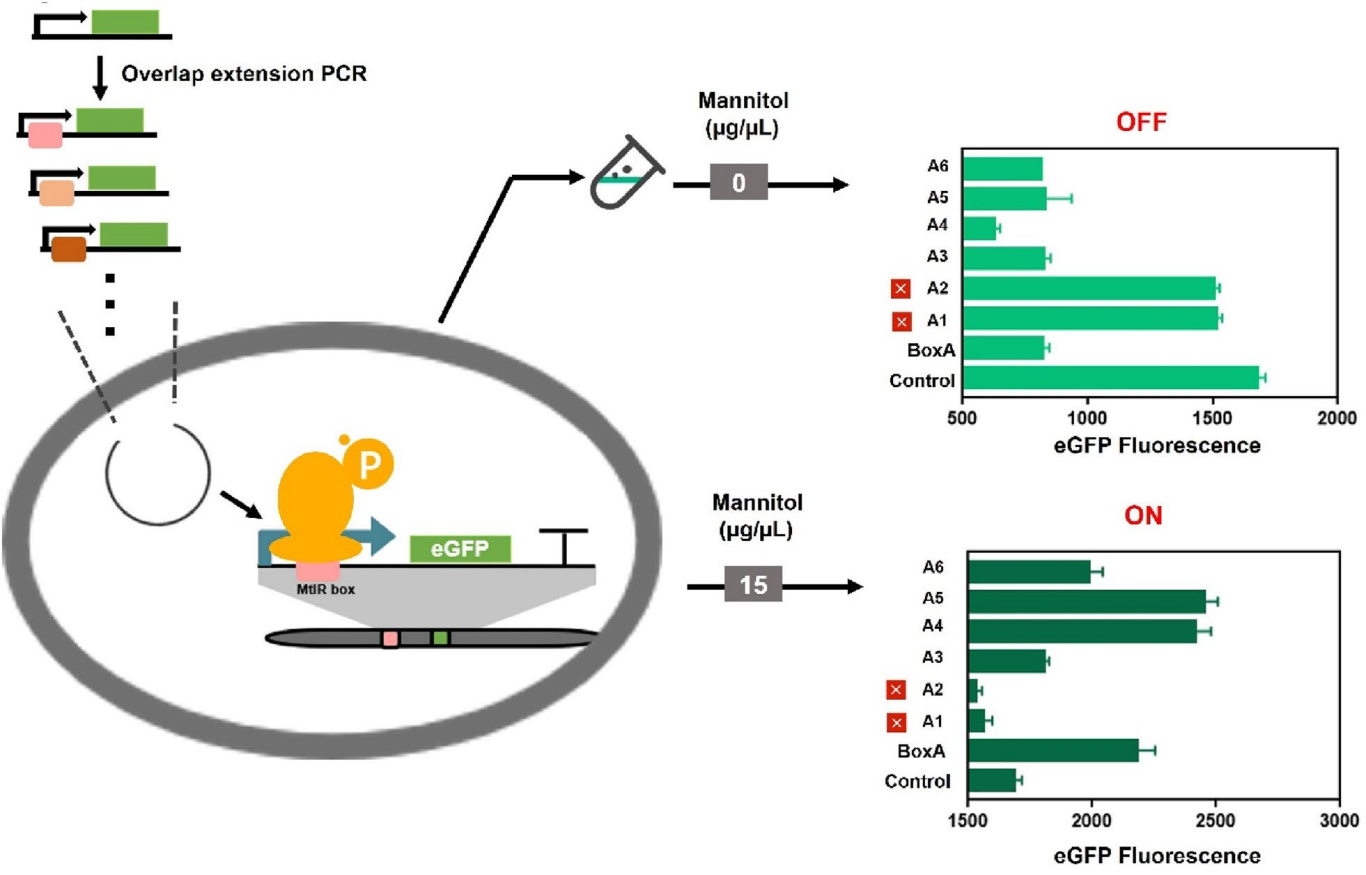
The rational design for MtlR box**.** The rational mutations were introduced into eGFP expression cassette by overlap extension PCR. Six MtlR boxes mutants were designed to test the OFF-state value and ON-state value. The resultant mutations were subjected to parallel operation of OFF/ON under two conditions (1) with no inducer (2) + 1.5% mannitol. The eGFP fluorescence was test after added inducer 12 h

**Fig. 3:**
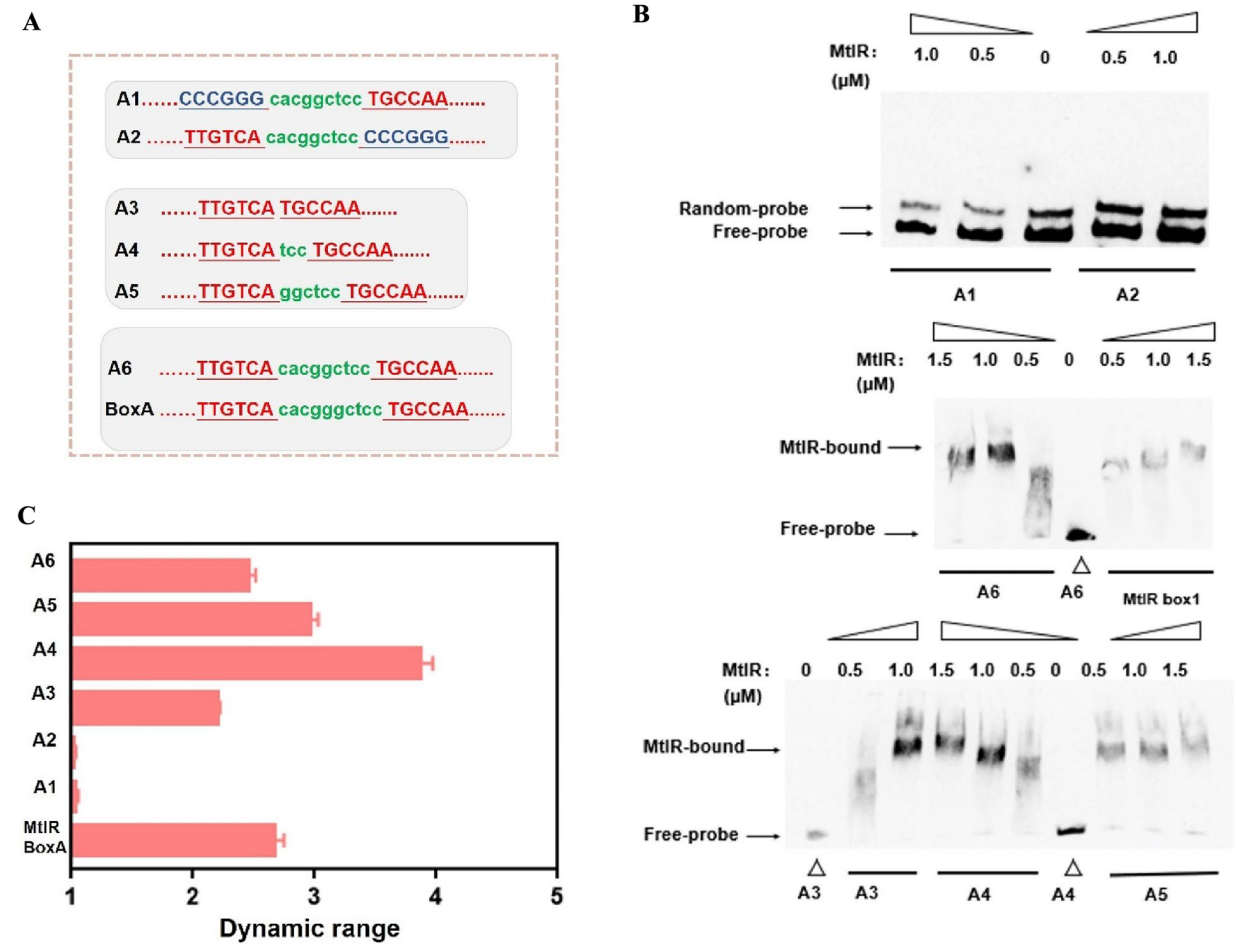
The test of MtlR box in vitro. **A** Gene sequences of different MtlR boxes. The sequence in red represent the conserved sequence of the MtlR box, and those in green represent non conserved sequence. MtlR box 1: CCCGGGcacggctccTGCCAA; MtlR box 2: TTGTCAcacggctccCCCGGG; MtlR box 3:TTGTCATGCCAA; MtlR box 4: TTGTCAtccTGCCAA; MtlR box 5: TTGTCAggctccTGCCAA;MtlR box 6: TTGTCAcacggctccTGCCAA. **B** Six fragments (A1, A2, A3, A4, A5, A6), that carried MtlR box 1, 2, 3, 4, 5, 6, respectively, were selected as probes, EMSA was performed by MtlR protein in different concentration (0, 0.5 µM, 1.0 µM) for six fragments. **C** The dynamic ranges of six MtlR boxes were shown

The sequence feature of the MtlR box is the core of genetic switch. Sequence alignment of MtlR box A and MtlR box B showed a same pseudo-palindromic sequence and a high ‘GC’ content sequence. Notably, the length of the box was uncertain due to the intermediate space region. This study aimed to design a series of mutations with different ON-state value and OFF-state value (Fig. 2). Based on the palindromic region, two mutation MtlR boxes (MtlR box 1, MtlR box 2) were designed (Fig. 3A). Two boxes were inserted separately into Pshutle09, thus generating two promoters named promoter A-1 and promoter A-2. First, we tested two boxes in vitro. When A-1 and A-2 were used as probes, no shifted band was observed. This indicated that MtlR had no affinity to MtlR box 1 or MtlR box 2 (Fig. 3B). This result also suggested that the pseudo-palindromic sequence was essential for MtlR binding. Next, we tested two boxes in vivo. The OFF-values of A-1 and A-2 were respectively decreased 9.79% and 10.39% in base-line and the ON-values of A-1 and A-2 were decreased 8.04% and 9.11% in maximum signal output, respectively. We have used random DNA instead of MtlR box to test whether the decrease in Pshutle09 strength was a coincidence (Additional file 1: Fig. S2). This indicated that random DNA would also decrease the strength of Pshutle09. Two artificial genetic circuits have a low dynamic range (1.03 × , 1.02 ×) (Fig. 3C), respectively. These results indicated MtlR box 1 and MtlR box2 were nonfunctioning as switch elements. Next, the effect of the intermediate spacer region of MtlR box was explored. Four MtlR boxes (MtlR box 3, MtlR box 4, MtlR box 5, and MtlR box 6) were designed (Fig. 3A). Four boxes were inserted separately into Pshutle09, thus generating four promoters, named promoter A-3, promoter A-4, promoter A-5, and promoter A-6. The OFF-values of A-3, A-4, A-5, and A-6 were respectively decreased 51.09%, 62.83%, 50.69%, and 51.67% in base-line and the ON-values were increased 7.19%, 43.35%, 45.41%, and 18.01% in maximum signal output, respectively (Fig. 2). The promoter A-4 that carried MtlR box 4 has a maximum dynamic range of 3.87 × (Fig. 3C). Then, we tested the affinity of MtlR for boxes 3, 4, 5, 6 in vitro. As shown in Fig. 3B, an obvious shifted band can be found when used A-3, A-4, A-5, A-6 as probes, indicating that the MtlR protein can bind to A-3, A-4, A-5, and A-6. These results suggested that the length of the intermediate spacer region was not a crucial determinant for MtlR binding. TF recognition sites that consist of a palindromic region and an intermediate spacer region were also found in other transcription factor, such as HipB and CcpA (Schumacher et al. 2015; Yang et al. 2017). This type of TF recognition sites is more feasible than the typical TF recognition site.

**Fig. 4:**
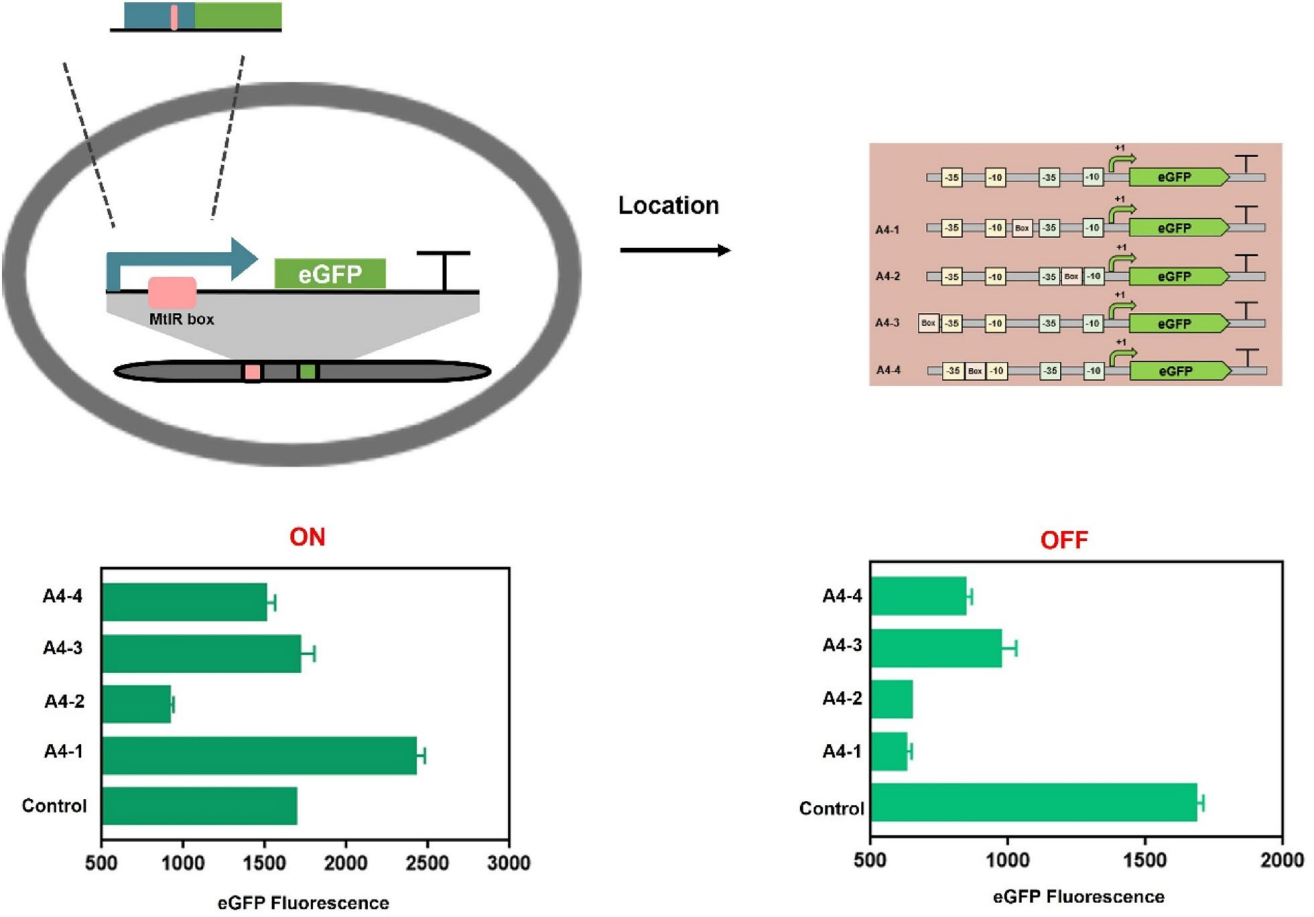
The influencing factor for MtlR box. Schematic diagram of integration of the MtlR box into different position of promoter Pshutle09, and the output values in ‘OFF’ and ‘ON’ state were shown

The hybrid genetic circuits based on the location of transcription factor binding site have been applied in synthetic biology. The insertion position of MtlR box in genetic circuit was also need to be tested. Considering MtlR box 4 has a higher dynamic range, it was selected as a follow-up study object. The promoter Pshutle09 has two − 10 region and − 35 regions, where those closer to the transcription start site are named − 10(1) region and − 35(1) region and those further from the transcription site are named − 10(2) region and − 35(2) region. As shown in Fig. 4, four artificial promoters were constructed with MtlR box in different locations. The MtlR box is located at the area between the − 10(2) region and the − 35(1) region in promoter A4-1 and the MtlR box is located at the area between the − 35(1) region and the − 10(1) region in promoter A4-2. The MtlR box is present in − 35(2) region in promoter A4-3, and the MtlR box is located between the − 10(2) region and the − 35(2) region in promoter A4-4. In the OFF-state, the values of A4-3 and A4-4 are higher than those of A4-1 and A4-2. The MtlR boxes in A4-3 and A4-4 were far from the transcription start site, indicating that the location of the MtlR box in the promoter also impacted the genetic circuit. The intensity of the four promoters increased to a different extent after adding mannitol, while A4-2 was significantly lower than the remaining three. Since A4-1 had higher ON value and lower OFF value, 4A-1 worked well comparing to the three remaining. As the RNA polymerase binding site located at this region, so it may influence the RNA polymerase binding when the MtlR box is located in this region.

Based on the ration design of the MtlR box, the dynamic range of the genetic circuit was expanded and the value of OFF-state was decreased. A potential MtlR box in the *B.licheniformis* genome was also investigated with the length of the intermediate spacer region decreasing by 10 bp. Nine potential MtlR boxes were found in the coding and non-coding regions within two boxes in the non-coding region and seven boxes in the coding region (Additional file 1: Table S5). These boxes involve genes that were engaged in different life activities, mostly involving protein transport. This result indicated that MtlR protein may be engaged in other life activities except mannitol metabolism. Designable TF DNA-binding is also one method for constructing complex dynamic regulatory circuits, since the affinity for the recognition site and the adaptation were changed (Cong et al. 2013; Gaber et al. 2014). However, the genetic circuit based on a single switch cannot respond to the multi-modular signals output, particularly in the aspect of rebalancing the central carbon metabolism and the product generated.

### Rational design of genetic circuits based on cre sites

**Fig. 5:**
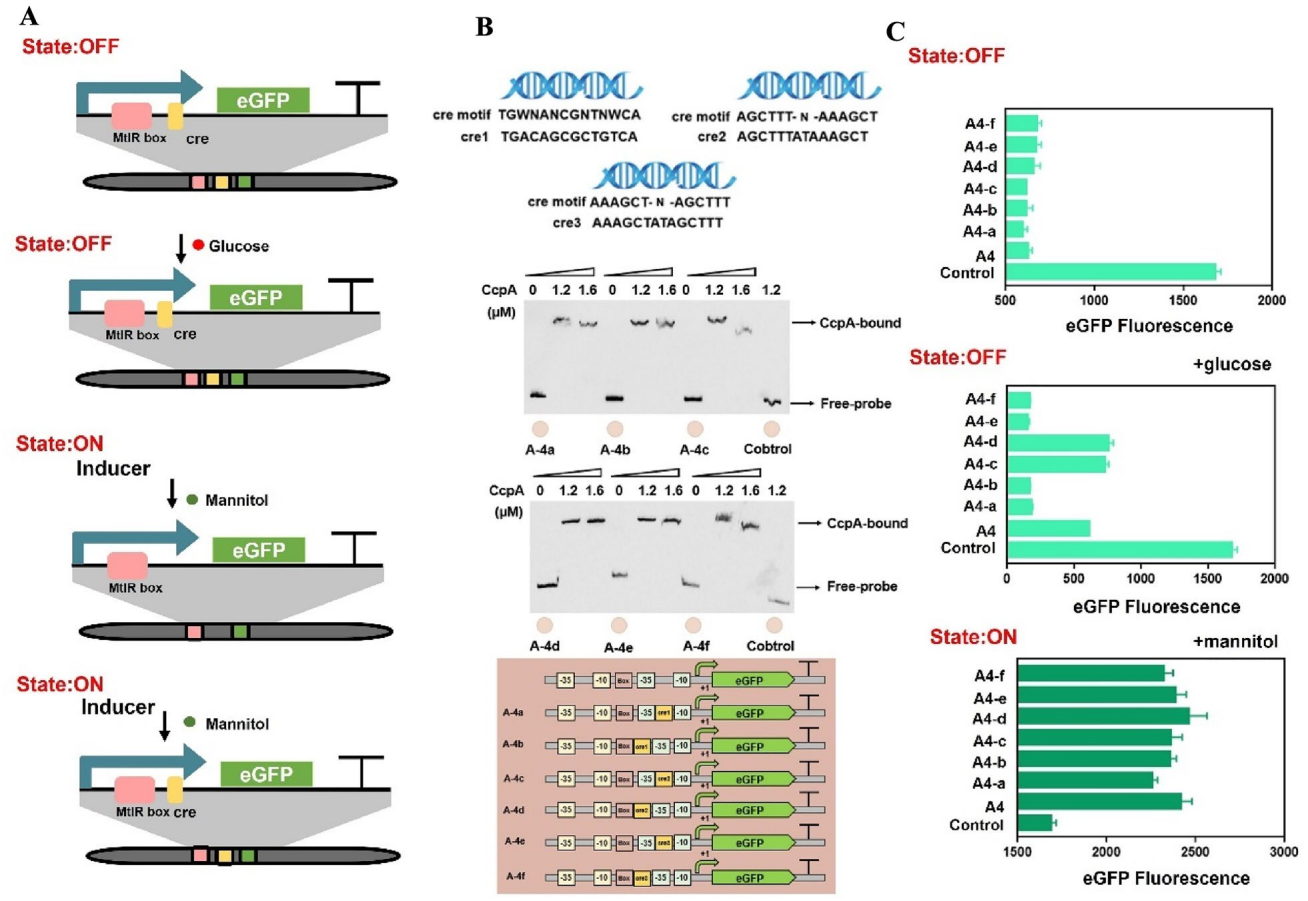
The test of MtlR box and cre in vivo and in vitro. **A** The state of genetic switch based on MtlR box and cre site. State OFF: MtlR box and cre site were added to the genetic circuit, and no substrate added. State OFF: MtlR box and cre site were added to the genetic circuit, and glucose added. State ON: MtlR box was added to the genetic circuit, and inducers added. State ON: MtlR box and cre site were added to the genetic circuit, and inducers added. **B** The construction of genetic circuit based on MtlR box and cre site. Three cre motifs is ‘TGWNANCGNTNWCA’, ‘AGCTTT-Yx-AAAGCT’, ‘AAAGCT-Yx- AGCTTT’ (in which N represents any base and W represents A or T, in which N represents any base and W represents A or T). Three cre sites were designed based on their motifs, in which cre1 sequence was ‘TGACAGCGCTGTCA’, cre2 sequence was ‘AGCTTTATAAAGCT’, cre3 sequence was ‘AAAGCTATAGCTTT’. Three cre sites were inserted to the artificial promoter with different location, generating six promoters ‘A-4a, A-4b, A-4c, A-4d, A-4e, A-4f’. Six fragments (A-4a, A-4b, A-4c, A-4d, A-4e, A-4f) were selected as probes, EMSA was performed by CcpA (0, 1.2, 1.6 µM) for six fragments. **C** The ON/OFF state values of the genetic circuit. The OFF-state values of six promoters ‘A-4a, A-4b, A-4c, A-4d, A-4e, A-4f’ when no substrate added. The OFF-state values of six promoters ‘A-4a, A-4b, A-4c, A-4d, A-4e, A-4f’ when glucose added. The ON-state values of six promoters ‘A-4a, A-4b, A-4c, A-4d, A-4e, A-4f’ when mannitol added

**Fig. 6:**
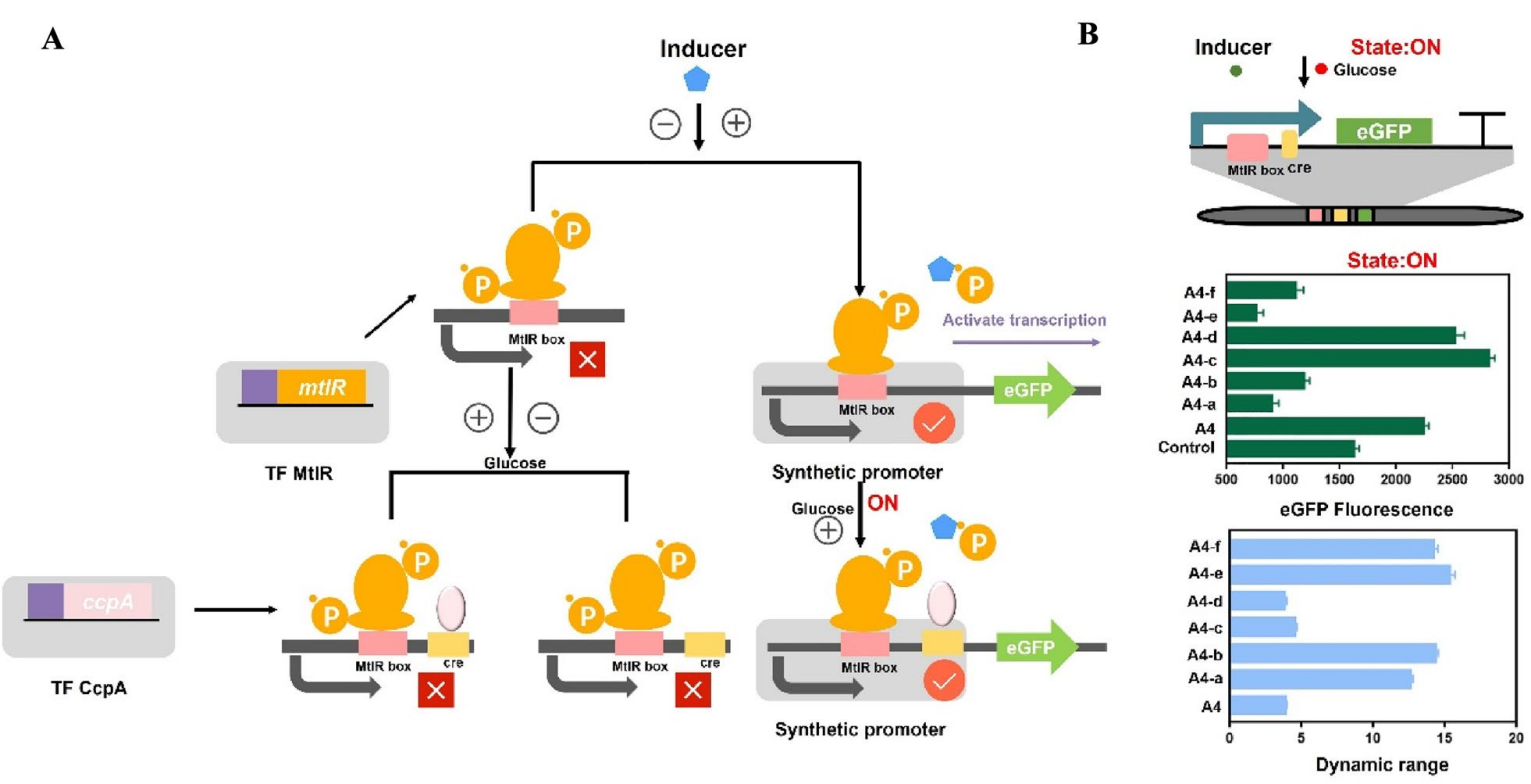
The test of MtlR box and cre in the presence of mannitol and glucose. **A** The MtlR box and mannitol determines the ON/OFF state of the genetic circuit, and the cre site expanded the dynamic range of the genetic circuit. **B** State ON: MtlR box and cre site were added to the genetic circuit, mannitol and glucose were added into the system. The dynamic range of six genetic circuits were also shown

In response to multi-modular signals output, we attempted to introduce cre site in genetic circuit for expanding its dynamic range. Current studies have confirmed that CcpA regulates gene expression by binding cre sites. Three types of cre sites have been found in *B.licheniformis*: ‘TGWNANCGNTNWCA’, ‘AGCTTT-N-AAAGCT’, ‘AAAGCT-N-AGCTTT’ (Xiao et al. 2021). Glucose can act as an effector for CcpA regulation. The cre site was added to genetic circuits to further decrease the OFF-value and increase the ON-value (Figs. 5A, 6A). Using the His-tagged protein kit, the CcpA protein that originated from *B.lichenifomis* was purified (Additional file 1: Fig. S3). We scanned the sequence of the promoter A-4 and no cre site was found. The EMSA result also confirmed it. No shifted band was observed when used A-4 as probes, which indicated that CcpA cannot bind to A-4 (Fig. 5B). Hence, the A-4 was selected as subject to carry the cre site. Usually, the cre site was located in the downstream of the specific promoter. For example, the cre site was located between the − 35 region and the − 10 region in the mannitol-inducible promoter P*mtlA*; the cre site was located at 7 bp upstream of the − 35 region in the trehalose-inducible promoter P*treA*; the cre site was located at 9 bp downstream of the -10 region in the rhamnose-inducible promoter P*rhaA* (Additional file 1: Fig. S4). Hence, two regions in A-4 (between the − 35 region and the − 10 region or 5 bp upstream of the − 35 region) were selected as the region for inserting the cre.

Based on the motif of the cre site, we designed three cre sites (Fig. 5B). Six promoters were constructed based on A-4 (A-4a, A-4b, A-4c, A-4d, A-4e, A-4f). First, we tested the CcpA’s affinity to cre sites in vitro. The CcpA protein was purified and the molecular weight was approximately 36.8 KDa. As shown in Fig. 5B, six obvious shifted bands were observed with A-4a, A-4b, A-4c, A-4d, A-4e and A-4f as probes, which indicated that CcpA can bind to these probes. Promoters A-4a and A-4b contain cre sites, which have a sequence of ‘TGACAGCGCTGTCA (cre1)’. Promoters A-4c and A-4d contain cre sites, which have a sequence of ‘AGCTTTATAAAGCT (cre2)’. Promoters A-4e and A-4f contain a cre site with a sequence of ‘AAAGCTATAGCTTT (cre3)’. When mannitol or glucose are not added, the genetic circuit is in the OFF-state and the values of the A-4a, A-4b, A-4c, A-4d, A-4e, A-4f were decreased by 64.60%, 63.30%, 63.37%, 60.91%, 60.05%, 59.93% in base-line, respectively. When glucose was added, the genetic circuit was also in the OFF-state and the values of the promoters A-4a, A-4b, A-4c, A-4d, A-4e, A-4f were decreased by 89.31%, 90.17%, 56.58%, 54.98%, 90.69%, 90.23% in base-line, respectively (Fig. 5C). Hence, A-4e (MtlR box 4, cre3) works well in the presence of glucose. These results indicated that cre1 and cre3 reduced the OFF-value of genetic circuits and this reduction was dependent on glucose and cre sites. Lower OFF-value is ideal for genetic circuit. In this study, a combination of two elements strategies were used to reduce the OFF-value and expand the dynamic range. These results confirmed that the two elements were independent on each other. When adding the mannitol, the genetic circuit was also in the ON-state and the values of A-4a, A-4b, A-4c, A-4d, A-4e, A-4f increased by 33.80%, 39.94%, 40.00%, 46.10%, 41.60%, 37.73% in maximum signal output, respectively (Fig. 5C). When the mannitol and glucose were added, the genetic circuits (A-4c, A-4d) were in the ON-state and the values of A-4c and A-4d increased by 73.34% and 55.23% in maximum signal output, respectively (Fig. 6B). Hence, A-4c (MtlR box 4, cre2) works well in the presence of mannitol and glucose. However, the values of the A-4a, A-4b, A-4e, A-4f decreased by 44.31%, 27.11%, 53.03%, 31.49% in maximum signal output in the presence of mannitol and glucose, respectively (Fig. 6B). A-4e (MtlR box 4, cre3) has a maximum dynamic range (15.31×). Therefore, a combination of the MtlR box and the cre site was an efficient method to expand the dynamic range of the genetic circuit.

Higher glucose tolerance and the ability of redirecting carbon flux from 2,3-butanediol to the target products provide an opportunity for the use of *B.licheniformis* in biotechnology (Xu et al. 2022). Glucose is the preferred carbon source for *Bacillus* sp. and it serves as a carbon substrate in the biosynthesis of a series of compounds. Typically, the specific genetic circuit is often required to produce of chemicals. Given the large amounts of glucose in the initial process, some elements that respond to glucose can be added to the genetic circuit, thus expanding the dynamic range of the genetic circuit. Carbon catabolite repression is an important global regulator mechanism. It ensures microorganism preferentially use a primary carbon source like glucose in complex carbon sources environments. Genes involved in transport and catabolism of secondary carbon sources are repressed until the primary carbon source is depleted. In this study, the cre site was added to the genetic circuit and it decreased the OFF-state value and expanded the dynamic range. After the cre1 and cre3 are inserted into the promoter, CcpA recognizes and binds to the sites that causes the CCR effect. When cre2 is inserted into the promoter, CcpA recognizes and binds to the site that causes the CCA effect. The study also found that the location of the cre site in the promoter also influenced the dynamic range. Previous studies have found the cre site was broadly distributed in the genome in *B.licheniformis*. The cre site can be found at the promoter region or coding region. Even in the promoter region, cre sites may exist different regions. This phenomenon provides the design idea for inserting the cre site to the target promoter. In addition to CcpA, there are also many global regulators in *Bacillus* such as CodY (Belitsky and Sonenshein 2011), which regulates the nitrogen metabolism; Spo0A (Mirouze et al. 2011), which participates in regulation of biofilm formation; TnrA (Yoshida et al. 2003), which regulates target gene expression under nitrogen-limited conditions; ComK (Leisner et al. 2007), which is important for the accumulation of mutations during stress conditions. These global transcription factors were not systemically explored in *B.licheniformis*. However, they all have the potential to develop as synthetic biology tools. With the development of synthetic biology, more tools are necessary for fine-regulation and dynamic regulation.

### Testing of the genetic switch using a reporter gene

**Fig. 7:**
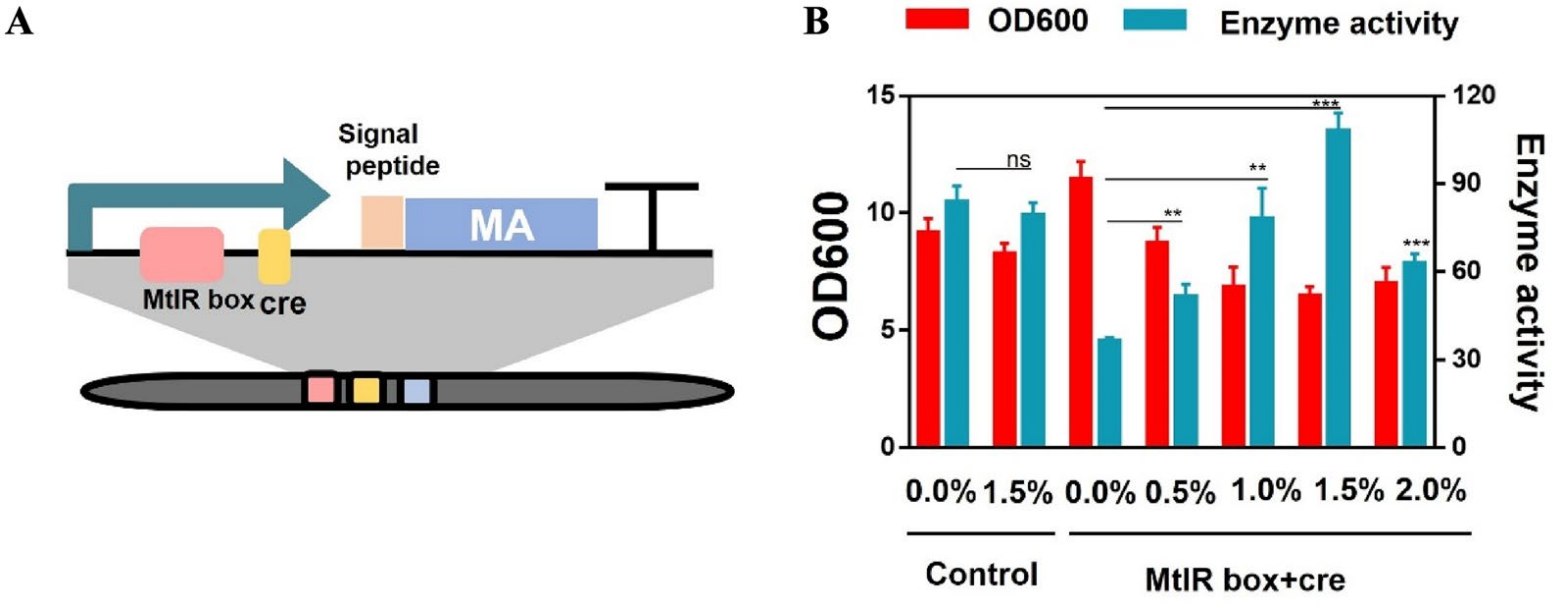
The test of genetic switch using a reporter gene. **A** The synthetic promoters were introduced into maltotriose amylase expression cassette. **B** The maltotriose amylase enzyme activity of the artificial promoter that carried MtlR box and cre in strains

At present, most biological applications strategies depend on growth-coupled production since constitutive expression of the target product is simpler than inducible expression. However, it may limit product yield and productivity. Specifically, some products are toxic to cells. In this study, two elements (the MtlR box and the cre site) were added to the same genetic circuit. The MtlR box and cre site were responsible for the ON-state and OFF-state. To further validate the function of the mannitol-responsive genetic circuit, maltotriose amylase was used as a reporter protein. The maltotriose amylase expression cassette was constructed for using an artificial promoter, the signal peptide from *B.subtilis* levansucrase, the maltotriose amylase gene and the terminator (Fig. 7A). First, a genetic circuit was constructed without a genetic switch. The circuit used Pshutle09 to guide maltotriose amylase gene persistent expression. The enzyme activity was measured at 85.89 U/mL at 18 h and the enzyme activity of the sample that added mannitol was measured at 78.56 U/mL, indicating that mannitol was not the effector for Pshutle09. Next, a genetic circuit was constructed with a mannitol switch-MtlR box and cre site, which was used promoter A-4c to control the maltotriose amylase gene expression. The OFF-state value was tested first and the enzyme activity was measured at 36.26 U/mL at 18 h with no mannitol added. This result indicated a decreased of 57.78% comparing to the sample that did not carry a genetic switch (Fig. 7B). These results suggested that the transcription of the target gene was part closure. At the same time, it also confirmed that the genetic switch performed the OFF-state function when no inducer added. The ON-state value was tested next and enzyme activity was measured at 108.72 U/mL at 18 h with 1.5% mannitol added, demonstrating a 199.84% increase comparing to the sample with no mannitol added. These results confirmed that the genetic switch was performed with the ON-state function when an inducer was added. The ON-state value was increased by 22.83% comparing to the genetic circuit that did not contain a genetic switch, which improved the width of the genetic circuit. Notably, the biomass of the sample with mannitol was lower than the sample that without mannitol (Additional file 1: Fig. S5). The influence of different concentrations of mannitol on the ON-state value was also tested. The ON-state values were tested with a concentration gradient (0.0%, 0.5%, 1.0%, 1.5%, 2.0%). The ON-state value was linearly related to the mannitol concentration (0–1.5%) and a linear fit was performed on the data points (*R*-square = 0.83241) (Additional file 1: Fig. S6). This result suggested that switch-based system could finely tune enzyme activity as well as controlling mannitol concentration. This allows more flexible operation of the genetic circuits. Additionally, a linear relationship was found for the mannitol switch within a certain range of mannitol concentration, meaning that the mannitol switch can apply to the biosensor. Such a short palindrome sequence-MtlR box controls the state of genetic circuits and can apply in different synthetic biology contexts.

## Conclusion

An ideal genetic circuit should have a switch element and a transcriptional regulatory element, which the switch element controls the genetic circuit in its ON-state or OFF-state and the transcriptional regulatory element controls the strength of genetic circuit. In this study, the MtlR box was selected as the switch element, and the cre site was chosen as the transcriptional regulatory element. Since the regulation effect of CcpA-cre is a bidirectional regulation, the genetic circuit is flexible. The uncontrolled genetic circuit was changed to the mannitol-inducible genetic circuit by adding a MtlR box and a cre site. In future studies, boxes based on other sugar operons should be explored such as mannose operon, arabinose operon. In addition, some transcription factors binding sites are worth studying as they can be used as transcription regulator tools. The combinations of different elements can be applied to different application scenarios. Moreover, the genetic circuit has great potential to control the expression of multi-gene biosynthetic pathways. Usually, coordinated gene expression is a vital method for the biosynthesis of high-value chemicals such as taxol (Jennewein et al. 2004). However, engineering microbial overproduction phenotypes remains a difficult problem (Xu et al. 2013). For example, enhancing precursor flux by heterologous pathways may not be accommodated, accumulated or depleted intermediates by downstream pathways may reduce cell viability (Leonard et al. 2010). Combinatorial genetic circuit coupled with efficient engineering transcription factor box may increase the adaptability of multi-gene pathways. In summary, this work enables the construction of high-performance genetic switches that have great potential for application in synthetic biology.

### Supplementary Information

**Additional file 1. Table S1.** Bacterial strains and plasmids used in this study. **Table S2.** The primers used in gene cloning and vectors construction. **Table S3**. The sequences of maltotriose amylase gene. **Table S4.** The sequences of artificial promoters. **Table S5.** The potential MtlR box in *B.licheniformis* genome. **Figure S1.** SDS-PAGE of purified MtlR protein. Figure S2. The test of random DNA for genetic circuit. **Figure S3.** SDS-PAGE of purified CcpA protein. **Figure S4.** The cre site in the native promoters. **Figure S5.** The cell growth curves of strain BlpSASMAT. **Figure S6.** The linear fit of mannitol concentration for enzyme activity.

## Data Availability

Data and materials described in this study are available from the authors upon reasonable request and availability.
