# Supplementary material for: Construction of the genetic switches in response to mannitol based on artificial MtlR box
Source: Bioresour Bioprocess. 2023 Jan 30;10(1):9. doi: 10.1186/s40643-023-00634-7 (PMC10992428; doi:10.1186/s40643-023-00634-7)

**Title:** Construction of the genetic switches in response to mannitol based on artificial MtlR box

Fengxu Xiao^a,b,c^, Yupeng Zhang ^a,b,c^, Liang Zhang ^a,b,c^, Zhongyang Ding ^a,b,c^, Guiyang Shi ^a,b,c^, Youran Li ^a,b,c*^.

^a^ Key Laboratory of Industrial Biotechnology, Ministry of Education, School of Biotechnology, Jiangnan University, Wuxi 214122, People’s Republic of China

^b^ National Engineering Research Center for Cereal Fermentation and Food Biomanufacturing, Jiangnan University, 1800 Lihu Avenue, Wuxi, Jiangsu Province 214122, People’s Republic of China

^c^ Jiangsu Provincial Engineering Research Center for Bioactive Product Processing, Jiangnan University

**Corresponding Author**:

Youran Li

*National Engineering Laboratory for Cereal Fermentation Technology, Jiangnan University, 1800 Lihu Avenue, Wuxi, Jiangsu Province 214122, People’s Republic of China*

e-mail: liyouran@jiangnan.edu.cn

Tel.: +86-0510-85918235

Fax: +86-0510-85918235

**Additional file 1**

# Content

Table S1 Bacterial strains and plasmids used in this study

Table S2 The primers used in gene cloning and vectors construction

Table S3 The sequences of maltotriose amylase gene

Table S4 The sequences of artificial promoters

Table S5The potential MtlR box in *B.licheniformis* genome

Figure S1 SDS-PAGE of purified MtlR protein

Figure S2The test of random DNA for genetic circuit

Figure S3 SDS-PAGE of purified CcpA protein

Figure S4 The cre site in the native promoters

Figure S5 The cell growth curves of strain BlpSASMAT

Figure S6 The linear fit of mannitol concentration for enzyme activity

Table S1 Bacterial strains and plasmids used in this study

| Strain or plasmid | Description | Reference |
| --- | --- | --- |
| Strains |  |  |
| *Escherichia coli*JM109 | F′, traD36, proAB + lacIq, Δ(lacZ), M15/Δ (lac-proAB), gln V44, e14−, gyrA96, | CICIM-CU |
|  | recA1, relA1, endA1, thi, hsdR17 (CICIM B0012) |  |
| *Bacillus licheniformis* | wild-type (CICIM B1391) | CICIM-CU |
| CICIM B1391 |  |  |
| *B. licheniformis* | *B. licheniformis* CICIM B1391, Δ*amyL*, Δ*aprE* (*amyL* encoding alpha amylase | CICIM-CU |
| CICIM B1522 | AmyL; *aprE* encoding subtilisin AprE) |  |
| BlpPSE | *B. licheniformis* CICIM B1391, harboring pPSE | This work |
| BlpPSAE | *B. licheniformis* CICIM B1391, harboring pPSAE | This work |
| BlpPSBE | *B. licheniformis* CICIM B1391, harboring pPSBE | This work |
| BlpPSA1E | *B. licheniformis* CICIM B1391, harboring pPSA1E | This work |
| BlpPSA2E | *B. licheniformis* CICIM B1391, harboring pPSA2E | This work |
| BlpPSA3E | *B. licheniformis* CICIM B1391, harboring pPSA3E | This work |
| BlpPSA4E | *B. licheniformis* CICIM B1391, harboring pPSA4E | This work |
| BlpPSA5E | *B. licheniformis* CICIM B1391, harboring pPSA5E | This work |
| BlpPSA6E | *B. licheniformis* CICIM B1391, harboring pPSA6E | This work |
| BlpPSA4C1E | *B. licheniformis* CICIM B1391, harboring pPSA4C1E | This work |
| BlpPSA4C2E | *B. licheniformis* CICIM B1391, harboring pPSA4C2E | This work |
| BlpPSA4C3E | *B. licheniformis* CICIM B1391, harboring pPSA4C3E | This work |
| BlpPSA4C4E | *B. licheniformis* CICIM B1391, harboring pPSA4C4E | This work |
| BlpPSA4C5E | *B. licheniformis* CICIM B1391, harboring pPSA4C5E | This work |
| BlpPSA4C6E | *B. licheniformis* CICIM B1391, harboring pPSA4C6E | This work |
| BlpSSMAT | *B. licheniformis* CICIM B1522,harboring pSSMAT | This work |
| BlpSASMAT | *B. licheniformis* CICIM B1522,harboring pSASMAT | This work |
| Plasmids |  |  |
| pHY300-PLK | *E. coli*/*Bacillus* shuttle vector, Ap^R^ /Tet^R^ | TaKaRa |
| pE | pHY300-PLK derivative with egfp | previous work |
| pPSE | pE derivative with promoter Pshutle09 | This work |
| pPSAE | pPSE derivative with MtlR BOX A (TTGTCACACGCTCCTGCCAA） | This work |
| pPSBE | pPSE derivative with MtlR BOX B (TTGTCACAGTCATGTGCCAA） | This work |
| pPSA1E | pPSE derivative with MtlR BOX 1 (CCCGGGCACGCTCCTGCCAA） | This work |
| pPSA2E | pPSE derivative with MtlR BOX 2 (TTGTCACACGCTCCCCCGGG) | This work |
| pPSA3E | pPSE derivative with MtlR BOX 3 (TTGTCATGCCAA） | This work |
| pPSA4E | pPSE derivative with MtlR BOX 4 (TTGTCATCCTGCCAA) | This work |
| pPSA5E | pPSE derivative with MtlR BOX 5 (TTGTCAGGCTCCTGCCAA） | This work |
| pPSA6E | pPSE derivative with MtlR BOX 6 (TTGTCACACGGCTCCTGCCAA) | This work |
| pPSA4C1E | pPSA4E derivative with cre1 between -35 region and -10 region | This work |
| pPSA4C2E | pPSA4E derivative with cre1 in 10 bp upstream of the -35 region | This work |
| pPSA4C3E | pPSA4E derivative with cre2 between -35 region and -10 region | This work |
| pPSA4C4E | pPSA4E derivative with cre2 in 10 bp upstream of the -35 region | This work |
| pPSA4C5E | pPSA4E derivative with cre3 between -35 region and -10 region | This work |
| pPSA4C6E | pPSA4E derivative with cre3 in 10 bp upstream of the -35 region | This work |
| pMA | pHY300-PLK derivative with maltotriose amylase gene (MA) | This work |
| pMAT | pMA derivative with terminator | This work |
| pSMAT | pMAT derivative with signal peptide | This work |
| pSSMAT | pSMAT derivative with promoter Pshutle09 | This work |
| pSASMAT | pSMAT derivative with promoter Pshutle09A4C3 | This work |

Ap^R^ ampicillin resistance, Tet^R^ tetracycline resistance, CICIM-CU Culture and Information Center of Industrial Microorganisms of China

Universities

Table S2

| Primers | Sequence | Restriction site |
| --- | --- | --- |
| Pshutle09-F | gaaaaacgctttgcccaagctttccccgtcagatggccgg | *Hin*dIII |
| Pshutle09-R | tggatccgcgacccatctcgagggatcccactttatggacgc | *Xho*I |
| SA-F | atataagcaaaaaactcttgtcacacgctcctgccaa |  |
| SA-R | aatgcttttcctttttatttggcaggagcgtgtgacaa |  |
| SB-F | atataagcaaaaaactcttgtcacagtcatgtgccaa |  |
| SB-R | aatgcttttcctttttatttggcacatgactgtgacaa |  |
| SA1-F | atataagcaaaaaactccccgggcacgctcctgccaa |  |
| SA1-R | aatgcttttcctttttatttggcaggagcgtgcccggg |  |
| SA2-F | atataagcaaaaaactcttgtcacacgctcccccggg |  |
| SA2-R | aatgcttttcctttttatcccgggggagcgtgtgacaa |  |
| SA3-F | atataagcaaaaaactcttgtcatgccaa |  |
| SA3-R | aatgcttttcctttttatttggcatgacaa |  |
| SA4-F | atataagcaaaaaactcttgtcatcctgccaa |  |
| SA4-R | aatgcttttcctttttatttggcaggatgacaa |  |
| SA5-F | atataagcaaaaaactcttgtcaggctcctgccaa |  |
| SA5-R | aatgcttttcctttttatttggcaggagcctgacaa |  |
| SA6-F | atataagcaaaaaactcttgtcacacggctcctgccaa |  |
| SA6-R | aatgcttttcctttttatttggcaggagccgtgtgacaa |  |
| SA4C1-F | tgacagcgctgtcatataatacaaaaagaccgattag |  |
| SA4C1-R | tgacagcgctgtcatttcaggtcaatgcttttccttt |  |
| SA4C2-F | tgacagcgctgtcaaaaagcattgacctgaaaacttat |  |
| SA4C2-R | tgacagcgctgtcacctttttatttggcaggatgaca |  |
| SA4C3-F | agctttataaagcttataatacaaaaagaccgattag |  |
| SA4C3-R | agctttataaagcttttcaggtcaatgcttttccttt |  |
| SA4C4-F | agctttataaagctaaaagcattgacctgaaaactta |  |
| SA4C4-R | agctttataaagctcctttttatttggcaggatgaca |  |
| SA4C5-F | aaagctatagcttttataatacaaaaagaccgattag |  |
| SA4C5-R | aaagctatagcttttttcaggtcaatgcttttcctt |  |
| SA4C6-F | aaagctatagctttaaaagcattgacctgaaaactta |  |
| SA4C6-R | aaagctatagctttcctttttatttggcaggatgaca |  |
| MA-F | ggggtaccatgggagtcagaagaagccttg | *Kpn*I |
| MA-R | cgcgtcgacctattatctccaagagtcgtaaaag | *Sal*I |
| T-F | cgcgtcgaccaggataagctccagat | *Sal*I |
| T-R | tcccccgggtaaaaaaccattcactct | *Sma*I |
| SP-F | cgcggatccatgaacatcaaaaagtt | *Bam*HI |
| SP-R | cggggtaccatgatgatgatgatgat | *Kpn*I |
| Pshutle09-F2 | cccaagctttccccgtcagatggccgg | *Hin*dIII |
| Pshutle09-R2 | cgcggatccggatcccactttatggac | *Bam*HI |

Table S3

|  | Sequence |
| --- | --- |
| MA | ATGGGAGTCAGAAGAAGCCTTGCAGCACTTCTTGCAGCACTTCTTGGCTGCGCAACGAGCCTGGTTGCACTTACAGTTGCAGCATCACCGGCTCATGCAGCACCTTCAGGAAACAGAGATGTCATCGTCCACTTATTTCAGTGGCGTTGGAAAAGCATCGCAGACGAGTGCAGAACGACATTAGGACCGCACGGATTTGGAGCAGTACAAGTTAGCCCGCCTCAAGAACACGTTGTCTTACCGGCAGAAGACTATCCTTGGTGGCAAGATTATCAGCCGGTCAGCTATAAGCTGGACCAAACAAGAAGAGGCAGCAGAGCGGACTTTATCGATATGGTCAACACGTGCAGAGAAGCAGGAGTCAAGATCTACGTCGATGCGGTCATCAACCACATGACAGGAACAGGATCAGCAGGAGCAGGACCGGGATCAGCAGGATCATCATATAGCAAGTACGACTATCCGGGCATCTATCAAAGCCAGGATTTCAACGATTGCCGCAGAGACATCACGAATTGGAACGACAAGTGGGAGGTCCAACATTGCGAACTTGTCGGACTGGCAGATCTTAAAACGAGCTCACCGTACGTCCAGGATAGAATCGCAGCGTATCTGAACGAACTGATCGACTTGGGAGTTGCAGGCTTTAGAATCGACGCAGCGAAACATATCCCGGAAGGAGATCTGCAAGCGATCCTGAGCAGACTGAAAAACGTCCATCCGGCTTGGGGAGGAGGAAAACCGTATATTTTCCAGGAAGTCATCGCGGATAGCACAATCAGCACAGGAAGCTATACACATCTGGGCTCAGTCACGGAATTTCAGTACCATCGCGACATCAGCCATGCATTTGCAAACGGCAACATCGCACATCTGACAGGACTTGGAAGCGGACTTACACCGTCAGATAAAGCAGTCGTGTTTGTCGTCAACCACGATACACAACGCTACGAACCGATCCTGACACATACAGACGGAGCAAGATACGACCTGGCACAGAAATTTATGCTGGCACATCCGTACGGCACACCGAAAGTCATGAGCAGCTATACCTGGTCAGGAGACGATAAAGCAGGACCTCCTATGCATAGCGACGGAACAACAAGACCGACAGATTGTAGCGCAGATCGTTGGCTTTGCGAACATAGAGCAGTCGCAGGAATGGTTGGATTTCATAACGCGGTCGCAGGACAAGGAATCGGATCAGCAGTTACAGACGGAAACGGAAGACTGGCATTTGCAAGAGGAAGCGCAGGATATGCAGCATTTAACGCGACAAACACAGCTTGGACGAGAACGTTTACGACAAGCCTTCCGGATGGAGTTTATTGCGATGTCGCGAACGGAACATTTGTCGATGGCGTTTGCGACGGACCTTCATATCAGGTCAGCGGAGGCAAATTTACGGCAACAGTTCCGGCAAACGGAGCAGTTGCACTTCACGTTGAAGCACCGGGATCTTGCGGACCTGACGGATGCGGAACACCTCCGGGAGGAGGAGACGATTGCACAACAGTCACGGCGAGATTTCACGCAACAGTTACCACCTGGTACGGACAAGAAGTTGCAGTTGTCGGAAGCATTCCGGAACTTGGATCTTGGCAACCGGCACAAGGAGTTAGACTGAGAACGGATAGCGGAACATATCCGGTTTGGTCAGGAGCAGTTGATTTACCGGCAGGCGTCGGCTTTGAATACAAGTACGTCAAGCTGAAACCGGACGGAACAGTTGAGTGGGAACAAGGAGGCAACAGAATCGCGACAGTTGATGATTCAGGAGGCGGCTGCTCACAAAACTTTTACGACTCTTGGAGATAA |

Table S4

| Pshutle09 | tccccgtcagatggccgggagccggatgaaccaccattccgcgcGgcttgttgacgacaagaacgtcctgatcttattataatataagcaaaaaactcataaaaaggaaaagcattgacctgaaAacttatcggtaaagtatgatataatacaaaaagaccgattagaggggagagaggaaacatgccttcagttgaaagttttgaacttgaccataatgcagtaaaagcgccttacgtcagacactgcggcgtccataaagtgggatccctcgagcgg |
| --- | --- |
| Pshutle09A | tccccgtcagatggccgggagccggatgaaccaccattccgcgcGgcttgttgacgacaagaacgtcctgatcttattataatataagcaaaaaactcttgtcacacggctcctgccaaataaaaaggaaaagcattgacctgaaAacttatcggtaaagtatgatataatacaaaaagaccgattagaggggagagaggaaacatgccttcagttgaaagttttgaacttgaccataatgcagtaaaagcgccttacgtcagacactgcggcgtccataaagtgggatccctcgagcgg |
| Pshutle09B | tccccgtcagatggccgggagccggatgaaccaccattccgcgcGgcttgttgacgacaagaacgtcctgatcttattataatataagcaaaaaactcttgtcacagtatgtgccaaataaaaaggaaaagcattgacctgaaAacttatcggtaaagtatgatataatacaaaaagaccgattagaggggagagaggaaacatgccttcagttgaaagttttgaacttgaccataatgcagtaaaagcgccttacgtcagacactgcggcgtccataaagtgggatccctcgagcgg |
| A-1 | tccccgtcagatggccgggagccggatgaaccaccattccgcgcGgcttgttgacgacaagaacgtcctgatcttattataatataagcaaaaaactcCCCGGGcacggctccTGCCAAataaaaaggaaaagcattgacctgaaAacttatcggtaaagtatgatataatacaaaaagaccgattagaggggagagaggaaacatgccttcagttgaaagttttgaacttgaccataatgcagtaaaagcgccttacgtcagacactgcggcgtccataaagtgggatccctcgagcgg |
| A-2 | tccccgtcagatggccgggagccggatgaaccaccattccgcgcGgcttgttgacgacaagaacgtcctgatcttattataatataagcaaaaaactcTTGTCAcacggctccCCCGGGataaaaaggaaaagcattgacctgaaAacttatcggtaaagtatgatataatacaaaaagaccgattagaggggagagaggaaacatgccttcagttgaaagttttgaacttgaccataatgcagtaaaagcgccttacgtcagacactgcggcgtccataaagtgggatccctcgagcgg |
| A-3 | tccccgtcagatggccgggagccggatgaaccaccattccgcgcGgcttgttgacgacaagaacgtcctgatcttattataatataagcaaaaaactcTTGTCATGCCAAataaaaaggaaaagcattgacctgaaAacttatcggtaaagtatgatataatacaaaaagaccgattagaggggagagaggaaacatgccttcagttgaaagttttgaacttgaccataatgcagtaaaagcgccttacgtcagacactgcggcgtccataaagtgggatccctcgagcgg |
| A-4 | tccccgtcagatggccgggagccggatgaaccaccattccgcgcGgcttgttgacgacaagaacgtcctgatcttattataatataagcaaaaaactcTTGTCAtccTGCCAAataaaaaggaaaagcattgacctgaaAacttatcggtaaagtatgatataatacaaaaagaccgattagaggggagagaggaaacatgccttcagttgaaagttttgaacttgaccataatgcagtaaaagcgccttacgtcagacactgcggcgtccataaagtgggatccctcgagcgg |
| A-5 | tccccgtcagatggccgggagccggatgaaccaccattccgcgcGgcttgttgacgacaagaacgtcctgatcttattataatataagcaaaaaactcTTGTCAggctccTGCCAAataaaaaggaaaagcattgacctgaaAacttatcggtaaagtatgatataatacaaaaagaccgattagaggggagagaggaaacatgccttcagttgaaagttttgaacttgaccataatgcagtaaaagcgccttacgtcagacactgcggcgtccataaagtgggatccctcgagcgg |
| A-6 | tccccgtcagatggccgggagccggatgaaccaccattccgcgcGgcttgttgacgacaagaacgtcctgatcttattataatataagcaaaaaactcTTGTCAcacggctccTGCCAAataaaaaggaaaagcattgacctgaaAacttatcggtaaagtatgatataatacaaaaagaccgattagaggggagagaggaaacatgccttcagttgaaagttttgaacttgaccataatgcagtaaaagcgccttacgtcagacactgcggcgtccataaagtgggatccctcgagcgg |
| A4-1 | tccccgtcagatggccgggagccggatgaaccaccattccgcgcGgcttgttgacgacaagaacgtcctgatcttattataatataagcaaaaaactcatTTGTCAtccTGCCAAaaaaaggaaaagcattgacctgaaAacttatcggtaaagtatgatataatacaaaaagaccgattagaggggagagaggaaacatgccttcagttgaaagttttgaacttgaccataatgcagtaaaagcgccttacgtcagacactgcggcgtccataaagtgggatccctcgagcgg |
| A4-2 | tccccgtcagatggccgggagccggatgaaccaccattccgcgcGgcttgttgacgacaagaacgtcctgatcttattataatataagcaaaaaactcataaaaaggaaaagcattgacctgaaATTGTCAtccTGCCAAtataatacaaaaagaccgattagaggggagagaggaaacatgccttcagttgaaagttttgaacttgaccataatgcagtaaaagcgccttacgtcagacactgcggcgtccataaagtgggatccctcgagcgg |
| A4-3 | tccccgtcagatggccgggagccggatgaTTGTCAtccTGCCAAaccaccattccgcgcGgcttgttgacgacaagaacgtcctgatcttattataatataagcaaaaaactcataaaaaggaaaagcattgacctgaaAacttatcggtaaagtatgatataatacaaaaagaccgattagaggggagagaggaaacatgccttcagttgaaagttttgaacttgaccataatgcagtaaaagcgccttacgtcagacactgcggcgtccataaagtgggatccctcgagcgg |
| A4-4 | tccccgtcagatggccgggagccggatgaaccaccattccgcgcGgcttgttgacgTTGTCAtccTGCCAAtattataatataagcaaaaaactcataaaaaggaaaagcattgacctgaaAacttatcggtaaagtatgatataatacaaaaagaccgattagaggggagagaggaaacatgccttcagttgaaagttttgaacttgaccataatgcagtaaaagcgccttacgtcagacactgcggcgtccataaagtgggatccctcgagcgg |
| A4-a | tccccgtcagatggccgggagccggatgaaccaccattccgcgcGgcttgttgacgacaagaacgtcctgatcttattataatataagcaaaaaactcTTGTCAtccTGCCAAataaaaaggaaaagcattgacctgaaATGACAGCGCTGTCAtataatacaaaaagaccgattagaggggagagaggaaacatgccttcagttgaaagttttgaacttgaccataatgcagtaaaagcgccttacgtcagacactgcggcgtccataaagtgggatccctcgagcgg |
| A4-b | tccccgtcagatggccgggagccggatgaaccaccattccgcgcGgcttgttgacgacaagaacgtcctgatcttattataatataagcaaaaaactcTTGTCAtccTGCCAAataaaaaggTGACAGCGCTGTCAaaaagcattgacctgaaAacttatcggtaaagtatgatataatacaaaaagaccgattagaggggagagaggaaacatgccttcagttgaaagttttgaacttgaccataatgcagtaaaagcgccttacgtcagacactgcggcgtccataaagtgggatccctcgagcgg |
| A4-c | tccccgtcagatggccgggagccggatgaaccaccattccgcgcGgcttgttgacgacaagaacgtcctgatcttattataatataagcaaaaaactcTTGTCAtccTGCCAAataaaaaggaaaagcattgacctgaaAAGCTTTATAAAGCTtataatacaaaaagaccgattagaggggagagaggaaacatgccttcagttgaaagttttgaacttgaccataatgcagtaaaagcgccttacgtcagacactgcggcgtccataaagtgggatccctcgagcgg |
| A4-d | tccccgtcagatggccgggagccggatgaaccaccattccgcgcGgcttgttgacgacaagaacgtcctgatcttattataatataagcaaaaaactcTTGTCAtccTGCCAAataaaaaggAGCTTTATAAAGCTaaaagcattgacctgaaAacttatcggtaaagtatgatataatacaaaaagaccgattagaggggagagaggaaacatgccttcagttgaaagttttgaacttgaccataatgcagtaaaagcgccttacgtcagacactgcggcgtccataaagtgggatccctcgagcgg |
| A4-e | tccccgtcagatggccgggagccggatgaaccaccattccgcgcGgcttgttgacgacaagaacgtcctgatcttattataatataagcaaaaaactcTTGTCAtccTGCCAAataaaaaggaaaagcattgacctgaaAAAAGCTATAGCTTTtataatacaaaaagaccgattagaggggagagaggaaacatgccttcagttgaaagttttgaacttgaccataatgcagtaaaagcgccttacgtcagacactgcggcgtccataaagtgggatccctcgagcgg |
| A4-f | tccccgtcagatggccgggagccggatgaaccaccattccgcgcGgcttgttgacgacaagaacgtcctgatcttattataatataagcaaaaaactcTTGTCAtccTGCCAAataaaaaggAAAGCTATAGCTTTaaaagcattgacctgaaAacttatcggtaaagtatgatataatacaaaaagaccgattagaggggagagaggaaacatgccttcagttgaaagttttgaacttgaccataatgcagtaaaagcgccttacgtcagacactgcggcgtccataaagtgggatccctcgagcgg |

Table S5

| **Gene** | **Location** | **Sequence** | **Length** | **Function** |
| --- | --- | --- | --- | --- |
| BaLi_c00720 | 70577-70597 | ttggcagcaagctcctgacaa | 21 | putative exporter YabM |
| BaLi_c03070 | 282017-282031 | ttgtcataatgccaa | 15 | D-galactarate dehydratase GarD |
| BaLi_c08310 | 817522-817540 | ttgtcattcccattgccaa | 19 | hypothetical protein |
| BaLi_c11650 | 1148486-1148504 | ttgtcaaccagtttgccaa | 19 | ABC transporter ATP-binding protein and permease YheI |
| BaLi_c13650 | 1349669-1349687 | ttgtcaggttttctgccaa | 19 | oligopeptide ABC transporter oligopeptide-binding protein OppA |
| BaLi_c21350 | 2065019-2065039 | ttgtcaataaatatttgccaa | 21 | amino acid carrier protein AlsT |
| BaLi_c21690 | 2102989-2103007 | ttgtcacttcagatgccaa | 19 | noyl-CoA hydratase/isomerase |
| BaLi_c40180 | 4006854-4006871 | ttgtcacaagattgccaa | 18 | ABC transporter permease compound YfiM |
| BaLi_c40990 | 4093719-4093737 | ttgtcattcccaatgccaa | 19 | transcriptional activator LicR |

**Figure S1**


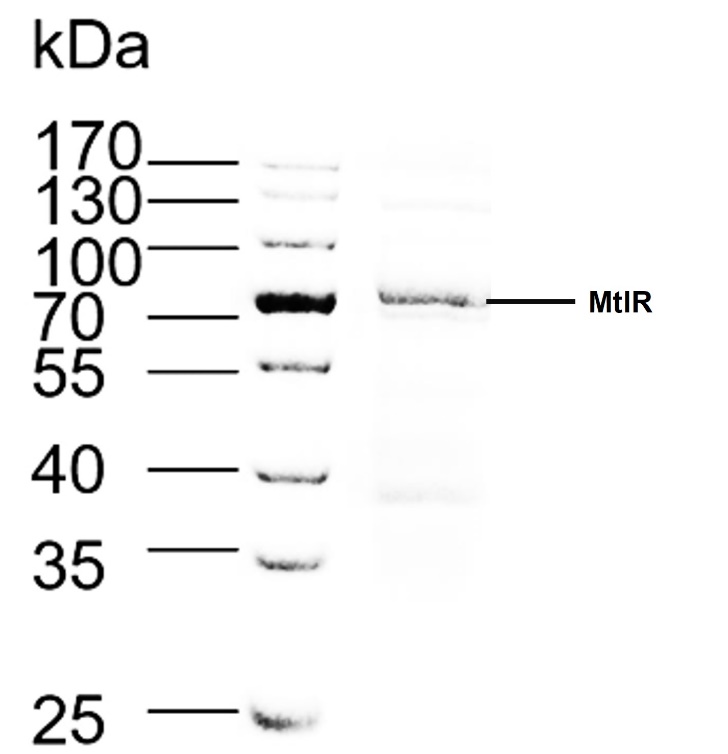


**Figure S2**


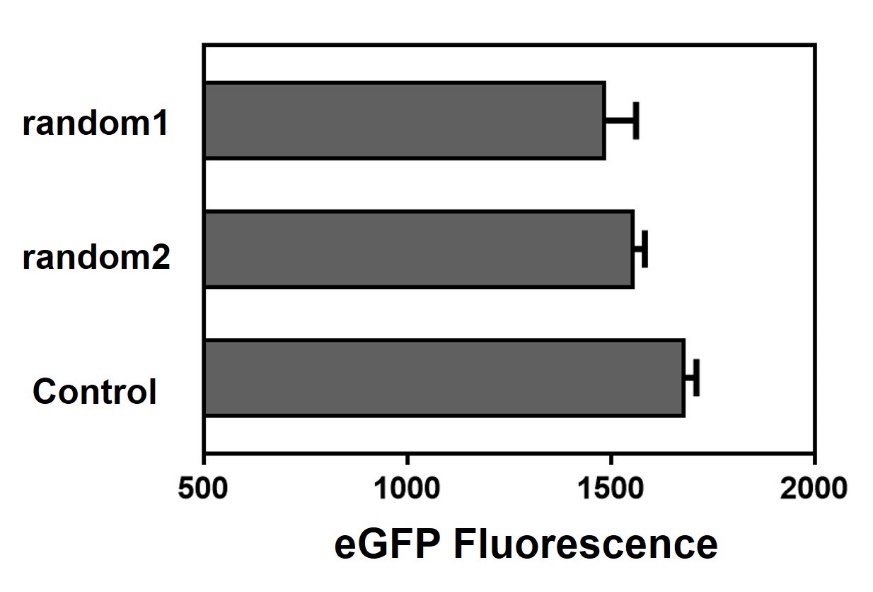


**Figure S3**


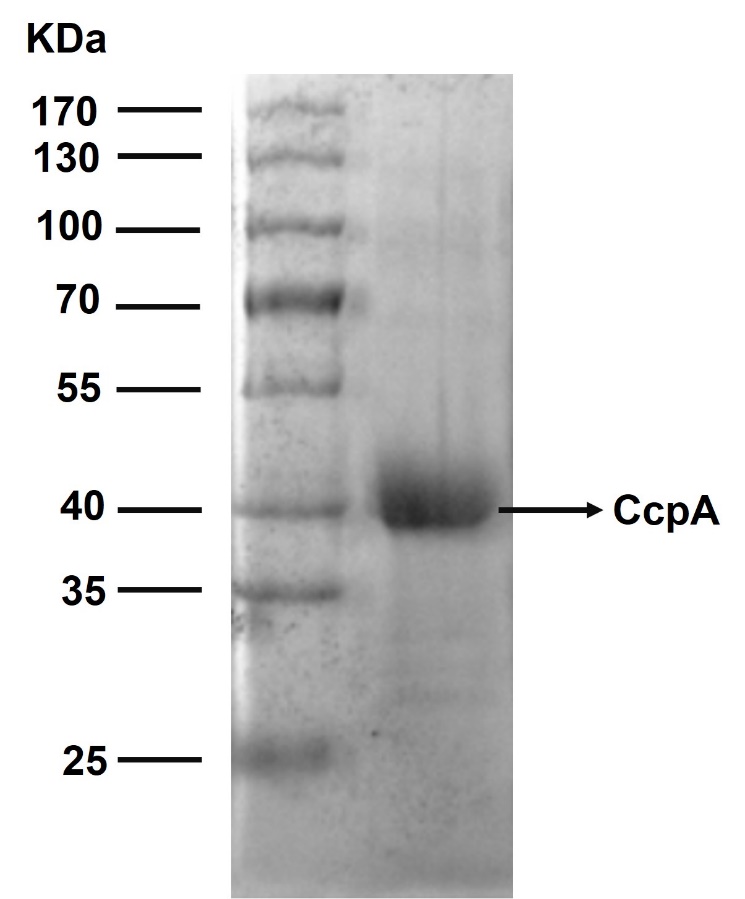


**Figure S4**


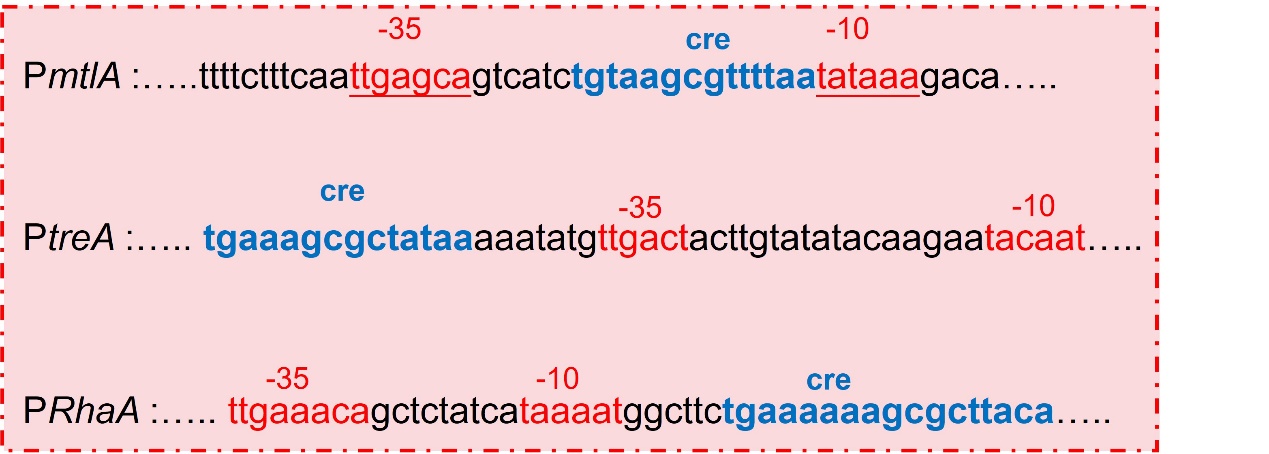


**Figure S5**


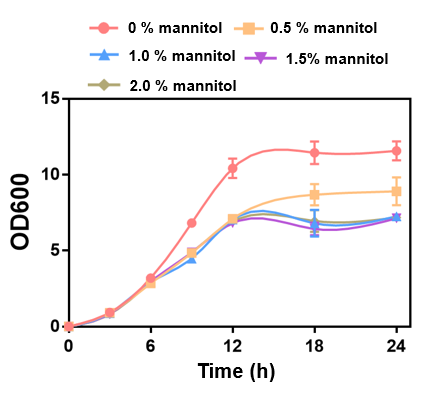


**Figure S6**


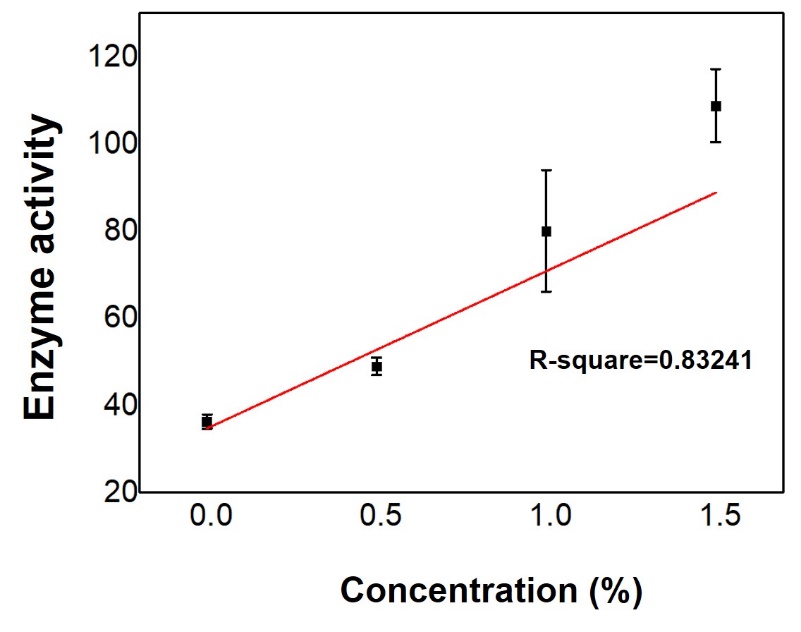

Supplement: Supplementary file 1 — Additional file 1. Table S1. Bacterial strains and plasmids used in this study. Table S2. The primers used in gene cloning and vectors construction. Table S3. The sequences of maltotriose amylase gene. Table S4. The sequences of artificial promoters. Table S5. The potential MtlR box in B.licheniformis genome. Figure S1. SDS-PAGE of purified MtlR protein. Figure S2. The test of random DNA for genetic circuit. Figure S3. SDS-PAGE of purified CcpA protein. Figure S4. The cre site in the native promoters. Figure S5. The cell growth curves of strain BlpSASMAT. Figure S6. The linear fit of mannitol concentration for enzyme activity. [file 40643_2023_634_MOESM1_ESM.docx]
